# Exploring the Potential of Overexpressed *OsCIPK2* Rice as a Nitrogen Utilization Efficient Crop and Analysis of Its Associated Rhizo-Compartmental Microbial Communities

**DOI:** 10.3390/ijms20153636

**Published:** 2019-07-25

**Authors:** Muhammad Umar Khan, Penghui Li, Hira Amjad, Ali Qaiser Khan, Yasir Arafat, Muhammad Waqas, Zhong Li, Ali Noman, Waqar Islam, Linkun Wu, Zhixing Zhang, Wenxiong Lin

**Affiliations:** 1 Fujian Provincial Key Laboratory of Agroecological Processing and Safety Monitoring, College of Life Sciences, Fujian Agriculture and Forestry University, Fuzhou 350002, China; 2 Key Laboratory of Crop Ecology and Molecular Physiology, Fujian Agriculture and Forestry University, Fuzhou 350002, China; 3 Key Laboratory for Genetics, Breeding and Multiple Utilization of Crops, Ministry of Education/College of Crop Sciences, Fujian Agriculture and Forestry University, Fuzhou 350002, China; 4 College of Agriculture, Nanjing Agricultural University, Nanjing 210095, China; 5 Department of Botany, Government College University, Faisalabad 38000, Pakistan; 6 College of Geographical Sciences, Fujian Normal University, Fuzhou 350007, China

**Keywords:** rice, microbiome, NUE, nitrogen, *CIPK2*, ion flux, transgenic, high-throughput sequencing

## Abstract

Nitrogen (N) is one of the indispensable factors in rice growth and development. China holds a premier position in the production of rice and at the same time also faces higher N fertilizer costs along with serious damage to the environment. A better solution is much needed to address these issues, without disrupting the production of rice as an important cereal, while minimizing all the deleterious effects on the environment. Two isogenic lines Kitaake (WT) and its genetically modified line CIPK2 (RC), overexpressing the gene for Calcineurin B-like interacting protein kinase 2 (Os*CIPK2*) with better nitrogen use efficiency (NUE), were compared for their growth and development under low versus normal levels of N. NUE is a complex trait mainly related to a plant’s efficiency in extraction, assimilation, and recycling of N from soil. The microbial population was analyzed using high-throughput Illumina Miseq 16S rRNA sequencing and found that RC with *CIPK2,* specifically expressed in rice root, not only performed better without nitrogen fertilizer (LN) but also increased the diversity of bacterial communities in rice rhizosphere compartments (rhizosphere, rhizoplane, and endosphere). The relative abundance of beneficial bacteria phyla increased, which are known to promote the circulation and transformation of N in rhizosphere soil. To further explore the potential of RC regarding better performance under LN, the ion fluxes in root apical were detected by non-invasive micro-test technique (NMT). We found that RC can absorb more Ca^2+^ and NO_3_^−^ under LN as compared to WT. Finally, compared to WT, RC plants exhibited better growth of root and shoot, and increased yield and N uptake under LN, whereas there was no significant difference in the growth of two rice lines under normal nitrogen (NN) treatment. We are able to get preliminary results, dealing with the *OsCIPK2* overexpressed rice line, by studying the rice molecular, physiological, and chemical parameters related to NUE. The results laid the foundation for further research on N absorption and utilization in rice from the soil and the interaction with microbial communities.

## 1. Introduction

Rice is one of the most important and major staple foods in the world. Therefore, the improvement of rice yield has always been a topic of concern and great interest around the world. With a continuously expanding world population, issues such as the shortage of water resources and the lack of arable land are becoming devastatingly serious. Hence, there is urgency to improve production by applying sustainable measures. At present, the most preferred technical measure to increase yield per unit area mainly depends on the application of fertilizers, especially nitrogenous ones [1,2]. Excessive N application rate and low NUE increase the production cost of rice, but also bring a series of environmental problems [3]. It is now particularly important to improve the nitrogen utilization efficiency (NUE) of rice and reduce the use of N fertilizers.

As N is the most essential nutrient in rice growth and development, the worldwide consumption of N-based fertilizers is approximately 119.40 million tons with an annual growth of 1.4% [4]. Asia consumes 62.1% of the total nitrogenous fertilizers and China alone shares 18% of the Asian N fertilizer consumption [4]. However, it is an alarming situation that major cereal crops like wheat, rice, and maize only utilize 30% to 40% of the applied N, while the remaining 60% to 70% poses severe health and environmental risks [5]. Compared with other major rice producing countries, the application rate of N fertilizers in China is high with a low utilization rate. Peng Shaobing and his group reported that the average amount of N fertilizer applied to rice in China has reached 180 kg/hm^2^, which was 75% higher than the world’s average level [6]. The goal of reducing the use of N-based fertilizers, improving the NUE, reducing the loss of N and its impact on the environment, on the premise of ensuring food security, is a critical problem to be solved in China and around the world.

Numerous strategies from agronomic level to transgenic improvements have been undertaken to solve this issue, including the split application of N, employing nitrification inhibitors, and the use of slow release fertilizers [7,8]. Although, conventional standard procedures such as selective breeding are helpful regarding the heritability of grain yield [9], all these remain far short of unravelling the genetic core for the improvements of intricate quantitative traits such as NUE [10]. Efforts are being made by scientists, such as the development of gene-overexpressed mutants to increase plant N contents in attempt to boost NUE in crop production [11,12]. At present, some genes involved in the regulation of N metabolism have been found in rice, including nitrate transporter gene (NRT family) [13], ammonium transporter gene (AMT family), nitrate reductase gene NR, nitrite reductase *NIR*, glutamine synthase (GS), glutamate synthase (GOGAT), etc., [14], and some genes indirectly involved in the regulation of N metabolism and N signal response. Some researchers regulate the expression of genes related to them through transgenic technology in an attempt to improve the NUE of rice, and have made some progress. A recent study revealed that the overexpression of *OsNRT1.1A* in different rice varieties and under different N conditions can significantly increase rice biomass and yield and significantly shortened the rice maturity period [15]. Fan Xiaorong and other studies also showed that overexpression of *OsNRT2.3b* could increase rice yield and NUE by 40% [16].

Similarly, CIPK (CBL-interacting protein kinase), a serine/threonine protein kinase, is a CBL (calcineurin B-like protein) protein-specific interacting protein kinase. CBL works as a calcium receptor protein, which was first found in *Arabidopsis thaliana*. As a second messenger of plants, Ca^2+^ participates in the response of plants to various signals. When plants are subjected to various stresses, intracellular calcium concentration changes [17]. Changes in intracellular Ca^2+^ concentration represent different stress signals, known as calcium signals; Ca^2+^ receptors can sense Ca^2+^ signals at any time, and then transmit signals through proteins, interacting with them, to regulate the expression of downstream response genes [18].

The CBL-CIPK signaling network is widely involved in abiotic stress responses, such as high salinity, high pH, low potassium, drought, low temperature, and abscisic acid [19]. *CIPK9* respond to multiple signals, such as to salt, low temperature, and osmotic stress, but also was significantly induced by low K^+^ stress [20]. At present, 10 CBLs and 33 CIPK genes have been found in rice, and Xiang has predicted and detected the changes of transcriptional levels of 30 *CIPK* genes in rice under drought, high salinity, cold, polyethylene glycol (PEG), and abscisic acid treatments [21]. However, it is not clear how the *CIPK* gene family participates in N response and regulation in rice, and further research is needed.

Another cardinal aspect to improve plant growth and yield involves studying the plant rhizospheric compartments, including rhizosphere soil (loosely attached soil to root), rhizoplane (soil surrounding the root surface), and endosphere (inside of the root). The process within plant roots, such as nutrient uptake, respiration, and root exudation, can significantly affect the chemical properties of rhizosphere soil and orchestrates the community structure of rhizosphere microorganisms [22].

The bacterial communities in plants and its rhizo-compartments impart its role in proper growth, as well as defense against biotic and abiotic stresses [23]. With modernization of microbial finger printing techniques, such as high-throughput sequencing, the plant–microbe relationship can be evaluated [24,25]. Soil and plant genotypes play a significant role in orchestrating the microbial community. Monocots are basically associated with the bacterial populations belonging to the Proteobacteria, Actinobacteria, Bacteroidetes, and Firmicutes, [26]. Chen Bin et al. used *nifH* gene to amplify nitrogen-fixing microorganisms, and combined with RFLP technology to study N-fixing microorganisms inside and outside rice root tissue. They found that there exist the same N-fixing microorganisms in rice rhizosphere and root tissue, indicating that some N-fixing microorganisms could colonize inside rice root or root surface [27]. Nitrification by bacteria plays an important role in regulating NH_4_^+^ and NO_3_^−^ levels in rhizosphere. Compared with single NH_4_^+^ or NO_3_^−^ nutrition, rice under mixed nitrogen nutrition could obtain more biomass and economic yield, with higher NUE rate [28].

Given these issues and indications, we developed isogenic lines from wild-type progenitor (Kitaake), involving the transgenic rice line overexpressing *CIPK2* gene, referred to as “RC” in this study. RC with overexpressing *CIPK2* gene performed significantly better than the wild type in NUE under LN (no nitrogen). The enzymes and genes involved in process N cycling were distinctly higher in the RC. Further, microbial communities were found to be much more diverse than the WT.

## 2. Results

### 2.1. Determination of CIPK2 Gene Expresion in Roots and Leaves of Transgenic and Wild-Type Rice

**Figure 1:**
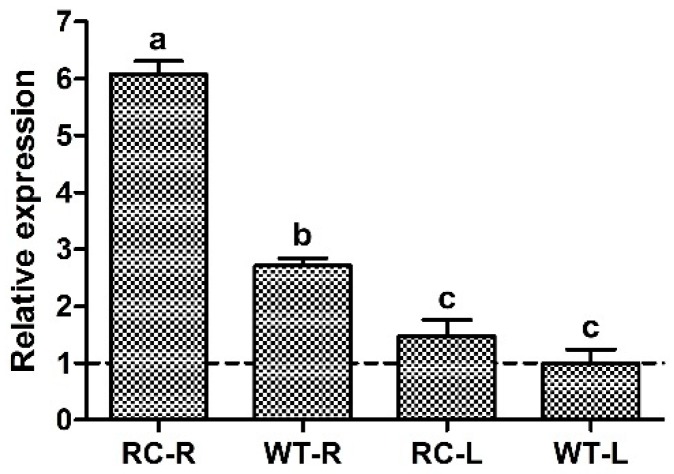
qPCR analysis of the gene expression level for Calcineurin B-like interacting protein kinase 2 (*CIPK2*). RC-R: Root of transgenic plants; WT-R: Root of wild type; RC-L: Leaf of transgenic plants; WT-L: Leaf of wild type. Small letters represent significant difference at (*p* < 0.05) calculated by Least Significance Difference (LSD) test.

The total RNA of roots and leaves of RC (transgenic line with overexpressed *OsCIPK2* gene) and WT (wild-type) rice showed a good integrity. OD absorbance ratio was set at 260:280 nm. We obtained 1.9–2.0, showing higher purity of RNA. The qPCR results revealed *CIPK2* gene expression was higher in rice roots than leaves. RC roots showed 2.24-fold Calcineurin B-like interacting protein kinase 2 (*CIPK2*) gene expression as compared to WT. While *CIPK2* expression in leaves was not significant (Figure 1).

#### Expression of CIPK2 Protein in Roots and Leaves of RC and WT Rice

**Figure 2:**
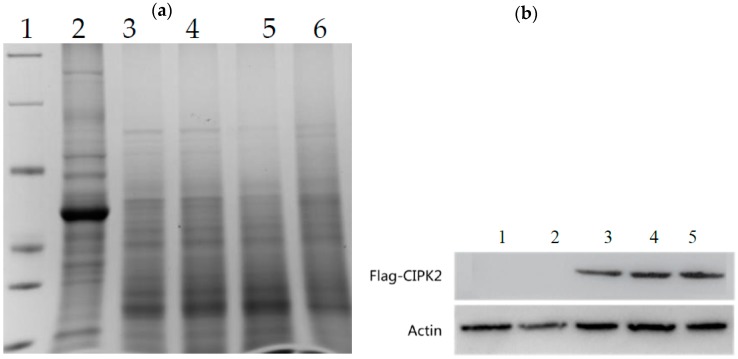
(**a**) SDS-PAGE test results of total protein from rice roots and leaves. Lane 1: Marker; Lane 2: RC (transgenic rice) leaves; Lane 3: WT (wild-type) roots; Lane 4–6: RC roots. (**b**) Western blot test results of Flag-CIPK2 protein in rice roots and leaves. Lane 1 RC leaves; Lane 2: WT roots; Lane 3–5: RC roots.

We employed western blot to further verify the root-type FLAG-CIPK2 fusion protein of RC, after getting clear protein bands (Figure 2a). We found the actin protein bands in each sample were clear and the expression levels were similar, indicating that the protein content of the sample loading is the same (Figure 2b). The FLAG-CIPK2 fusion protein was not detected in the WT rice roots and in the RC leaves.

### 2.2. Bucket Experiment to Determine Differences between WT and RC

#### 2.2.1. Changes in Tiller and Panicle

The tillering dynamics of rice under different N conditions are shown in Figure S1a. Under normal nitrogen (NN) conditions, the tillering number of rice increased continuously, while the tillering number decreased at maturity. Under LN, tillering mainly occurred in the early stage, but in the later stage, due to insufficient N supply, tillering no longer increased. From Figure S1b it can be seen that the panicle-forming rate of rice was higher under the condition of LN, the number of effective panicles of RC rice was significantly higher than the WT. There was no significant change in the effective panicles of RC rice under NN condition, but the panicle-forming rate was significantly reduced due to the increase of ineffective tillers.

#### 2.2.2. Chlorophyll (SPAD Values) and Changes in Photosynthetic Rate

**Figure 3:**
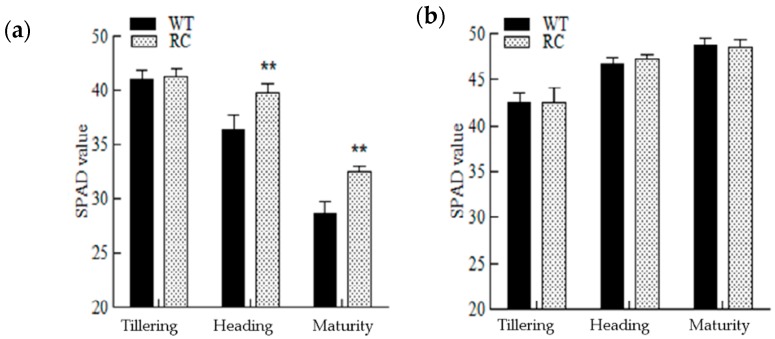
SPAD values of rice leaves at different growth stages under different treatments in pots: (**a**) No nitrogen treatment—LN; (**b**) normal nitrogen treatment—NN. ** Indicates significant difference (*p* < 0.05) between different samples in the same growth period calculated by LSD’s test.

**Figure 4:**
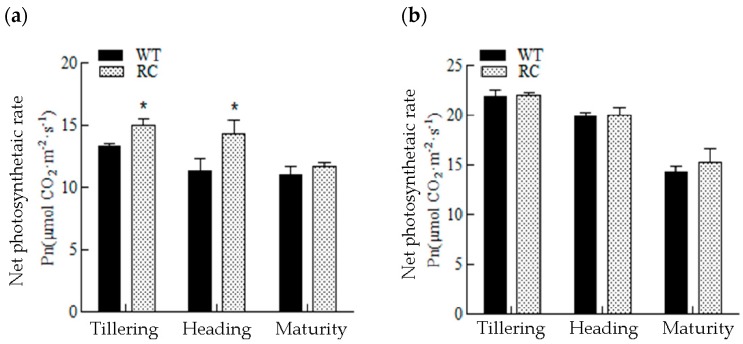
Photosynthetic rate of rice leaves at different growth stages under different nitrogen treatments in pots: (**a**) No nitrogen treatment—LN; (**b**) normal nitrogen treatment—NN. * Indicates significant difference (*p* < 0.05) between different samples in the same growth period, calculated by LSD’s test.

From Figure 3, it can be seen that under LN, the SPAD (soil–plant analyses development) meter readings, for determining chlorophyll content of rice leaves, gradually decreased with the development of rice. Compared with the WT, the SPAD values of RC leaves at tillering stage was not significantly different, but the SPAD values of RC leaves at heading stage and maturity stage was significantly higher than that of the WT. Under NN conditions, soil N was sufficient, SPAD values of rice leaves increased gradually, and there was no significant difference between RC and WT. The net photosynthetic rate (Figure 4) of RC at tillering and heading stages was significantly higher than that of wild type, but there was no significant difference at maturity stage, and there was no significant difference at all stages under NN.

#### 2.2.3. Changes in Root Morphology

The root morphology was analyzed by WinRHIZO (Instruments Regent Inc., Sainte-Foy, QC, Canada) at the main growth stages of rice. We observed that the length of adventitious roots, branched roots, the surface area of total roots, and the volume of total roots of RC at the heading stage were significantly higher than WT under the LN condition. The length of adventitious roots and the length of branched roots of RC at maturity stage were significantly higher than WT because of senescence of roots. Under NN conditions, the root morphological characteristics of the two lines did not change significantly. Interestingly, the roots of RC were more developed under LN (Table S2). Similarly, Figure S2 also depicts that the roots of RC were obviously more developed than those of WT.

#### 2.2.4. Root Activity

The root activity of rice was measured during the main growth periods of rice. The results are shown in Figure S3. Under the condition LN, the root activity of RC significantly increased in each period, and the root activity was highest at the heading stage, and the difference was significant. Under NN application conditions, there was no significant difference at each growth stage.

#### 2.2.5. Changes in Nitrogen-Related Enzyme Activities in WT and RC

**Figure 5:**
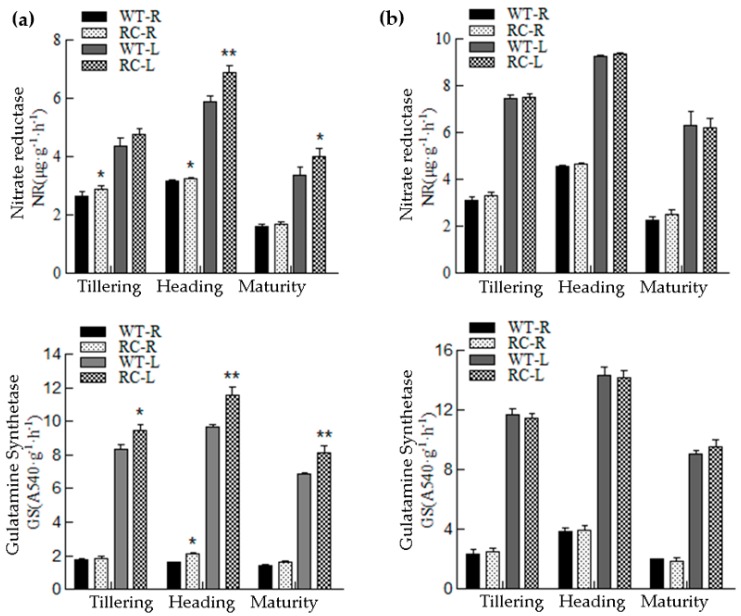
Nitrate reductase and glutamine synthetase activity of rice at different growth stages under different nitrogen treatments in pots. WT-R: Root of wild type; RC-R: Root of transgenic rice; WT-L: Leaf of wild type; RC-L: Leaf transgenic rice. (**a**) No nitrogen treatment—LN; (**b**) normal nitrogen treatment; NN. *, ** represent significance difference (*p* < 0.05) calculated by LSD’s test.

Nitrate reductase (NR) and glutamine synthetase (GS) are the key enzymes involved in the process of N assimilation. From Figure 5 we can see that NR and GS in the leaves and roots of RC were higher than those of WT at all growth stages under LN condition, and the enzymatic activity of leaves increased significantly. There was no significant difference under NN conditions.

#### 2.2.6. Changes in Dry Matter Weight and Root:Shoot Ratio

The root dry weight and shoot dry weight of rice at different stages were investigated and the root:shoot ratio was calculated. The results showed (Figure S4) that under the condition of LN there was no difference in dry matter weight between RC and WT at the tillering stage. Root dry weight and shoot dry weight at heading and maturing stages were significantly increased, shoot dry matter weight at maturing stage was significantly increased, and root:shoot ratio of RC at heading stage was significantly increased. Under NN conditions, there was no significant difference found.

#### 2.2.7. Changes in Yield and Yield Components

The yield and yield components of rice (Table 1) under LN showed that yield of rice was significantly lower than that of NN. In terms of yield components, the effective panicles of both RC and WT rice were significantly reduced under the LN treatment. Although the number of panicles and grains of WT were significantly reduced, the seed setting rate increased significantly. The change in panicles and grains of RC was not obvious, but the seed setting rate significantly decreased. The change of 1000 grain weight was also not obvious. Under LN application, the yield of RC increased by 35% as compared to WT, mainly due to the increase of panicles. Under NN, the panicle-forming rate of RC decreased, but other differences were not significant.

#### 2.2.8. Changes in Nitrogen Use Efficiency

Compared with the WT, the N uptake of RC increased by 38.51% in LN, the N dry matter production efficiency and grain production efficiency did not change significantly, the N harvest index decreased significantly, and the difference was not significant under NN application conditions (Table 2.). Under NN, the physiological, agronomic, and absorption utilization rates of RC were significantly reduced.

### 2.3. Changes in Bacterial Communities in Rhizosphere, Rhizoplane, and Endosphere of Rice Expressing CIPK2 with Enhanced Root-Specific Expression

#### 2.3.1. Analysis of Bacterial Community Diversity

Firstly, the number of operational taxonomic unit (OTUs) detected in each sample was analyzed. The results showed that the number of OTUs in rhizosphere soil (R), rhizoplane (RP), and endosphere (ES) decreased under different NN levels (Figure S5.). Under the LN condition, there was 2506, 2444, and 1164 OTUs in RC and 2435, 2331, and 1263 OTUs identified in WT. Under NN conditions, RC was identified with 3015, 2874, and 1149 OTUs, and WT with 2934, 2921, and 1331 OTUs, respectively. The OTUs number of R and RP under NN treatment was significantly higher than that under LN treatment, but the difference of OTU number in ES was not significant and the ES OTU number in root was significantly lower than that in R and RP. Among the RC and WT, 94.04% and 96.03% of RP OTUs belonged to R and ES, 97.42% and 96.91% of ES OTUs belonged to R and RP, respectively. Under N treatment, the trend was the same, indicating that most of the microorganisms in RP and in the ES were the same as those in the R.

The diversity of individual samples (alpha diversity) was further analyzed by 97% similarity. Observed_species is the number of observed OTUs with the increase of sequencing depth. As shown in Table 3, the observed_species value, Chao1 index, and PD_whole_tree value of rhizosphere bacterial community of RC were significantly higher than those of WT, while observed_species value and PD_whole_tree value of RP bacterial community of RC were significantly higher than those of WT. There was no significant difference in the alpha diversity index of bacterial community between the two types. Which depicts that the diversity of bacterial communities in R and RP of RC was higher than WT, but there was no significant difference in bacterial diversity in ES. Under NN treatment, the diversity index of bacterial communities in R, RP, and ES of rice was higher than that of LN treatment, but there was no significant difference between RC and WT. By comparing the alpha diversity index of bacterial community in different rhizo-compartments of the same sample, it was found that the diversity index of bacterial community in R was higher than the RP, and the diversity index of bacterial communities in ES was significantly lower than that in in other rhizo-compartments, a similar trend under different NN treatments.

#### 2.3.2. PCA Analysis of Rhizosphere Bacterial Community in Rice

In order to reflect the relationship between bacterial community distributions in rhizo-compartment, PCA analysis was carried out using OTUs with 97% similarity. Figure S6a is the PCA analysis results of bacterial communities in all samples with LN application. The first axis explains 74.57% of the species change, and the second axis explains 9.52% of the species change. PCA shows the differences between bacterial communities in all rhizo-compartments. In comparison with WT, obvious differences between the microbial communities in rhizo-compartments of RC were found. Figure S6b is the PCA analysis result under NN treatment. The first axis explains 72.24% of species change and the second axis explains 6.14% of species change. Under N conditions, bacterial communities in all rhizo-compartments could be distinguished. There was also a distinction between R and RP bacterial communities in RC and WT, but ES of both RC and WT bacterial communities of RC were not significantly different.

#### 2.3.3. Analysis of Bacterial Community Composition

**Figure 6:**
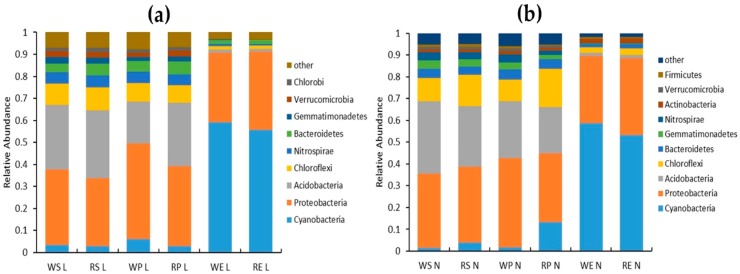
The main phyla of bacteria in rhizo-compartments of rice (**a**) under LN—no nitrogen; and (**b**) under NN—normal nitrogen.

Thirty-nine bacterial phyla were identified in LN treatment, of which Cyanobacteria, Proteobacteria, Acidobacteria, Chloroflexi, Nitrospirae, Bacteroides, Gemmatimonadetes, Verrucomicrobia, and Chlorobi account for more than 94% of the total (Figure 6a). The main bacterial phyla in ES were Cyanobacteria and Proteobacteria. The relative abundance of Cyanobacteria was 55% and 30% of Protobacteria. The abundance of other phyla in ES was significantly lower than that in R and RP, which also depicts the bacterial diversity in roots was significantly lower than other rhizo-compartments. The main bacterial phyla in R and RP were similar consisting mainly of Proteobacteria and Acidobacteria, accounting for more than 60% of the total bacterial phyla. Among them, Protobacteria was the highest in abundance, and the RP relative abundance was higher than that in R and ES. From RP to rhizoplane, the abundance of Chloroflexi, Gemmatimonadetes, and Chlorobi decreased, but there was no significant difference among other phyla. Compared with the WT, the abundance of rhizosphere Bacteroides and Chlorobi increased. The rhizoplane Gemmatimonadetes and Verrucomicrobia increased while Proteobacteria decreased. The abundance of Proteobacteria decreased significantly in rhizo-compartments of RC.

A total of 43 bacterial taxa were identified under NN conditions. The main bacterial phyla were identical with those under LN application (Figure 6b). Compared with LN treatment, the abundance of Nitrospirae decreased, the abundance of Verrucomicrobia decreased. While the abundance of Chlorobi, Actinobacteria, and Proteobacteria increased. The abundance of Chloroflexi and Cyanobacteria in RC was higher than that in WT, while the abundance of Acidobacteria was lower and the rest of the bacteria phyla did not change significantly.

#### 2.3.4. Bacterial Functional Analysis

We used function annotation database FAPROTAX [29] of prokaryotic microorganisms to predict the function of all bacterial classifications detected. A total of 36 types of bacterial functions were obtained, and different bacterial functions and relative abundance are shown in Figure S7. The main functions include nitrogen cycle, iron oxidation and reduction, organic matter degradation, chemotrophic, and phototrophic, among which R and RP had the highest bacterial abundance of chemoautotrophic bacterial population, followed by bacterial communities involved in nitrification. Compared with WT, the abundance of bacteria involved in nitrification and N fixation increased, and mainly concentrated in R and RP, while the abundance of bacteria involved in denitrification decreased. In the R and RP of RC, the bacterial abundance involved in the reduction of iron, sulfur, fumaric acid, manganese, and other related compounds decreased, while the bacterial abundance involved in the oxidation of iron and manganese increased. The ES bacterial abundance of RC involved in chitin decomposition, cellulose, and lignin degradation increased, while the bacterial abundance associated with carbohydrate degradation decreased. The abundances of iron oxidizing, phototrophic, and heterotrophic bacteria in RC roots were significantly increased. Interestingly, the abundances of pathogenic bacteria in all rhizo-compartments decreased under LN.

Compared with LN treatment, the abundance of bacterial communities involved in nitrification, denitrification and N fixation decreased in all rhizo-compartments under NN treatment. Under NN conditions, the bacterial abundance related to iron reduction process increased, but the bacteria involved in iron oxidation process were not identified. The abundance of bacteria involved in chitin, cellulose, and urea decomposition in R and RP increased, while the abundance of pathogens decreased. Compared to WT, the bacterial abundance involved in photosynthesis increased significantly in R and in RE of RC, while the bacterial abundance involved in chitin decomposition decreased, contrary to the trend of NN application. Compared with WT, most of the bacterial functions of RC did not change significantly under NN treatment, and the changes were more obvious under LN application.

#### 2.3.5. qPCR Verified the Changes of Genes Related to Rhizosphere Nitrogen Cycle under LN

In order to further verify the changes of nitrogen cycle-related bacteria in rice rhizosphere without nitrogen application, the nitrogen cycle genes *nifH, narG, nirK, nirS, nosZ, amoA* (AOA)*, amoA* (AOB) in rhizosphere soil were quantified by qPCR. The results are shown in Table 4. In the rhizosphere soil of RC, the number of copies of *nifH*, *amoA,* and *nirS* genes involved in ammonia oxidation and denitrification increased significantly, while the copy number of *nirK* gene decreased significantly, and the copy number of *narG* and *nosZ* genes did not change significantly. The results further confirmed the increase of ammonia-oxidizing bacteria and nitrogen-fixing bacteria in the rhizosphere soil of RC, indicating that the nitrogen cycle in RC was further enhanced.

### 2.4. Changes in Soil N-Related Physicochemical Properties under LN

Soil enzymes are involved in the transformation of many substances and nutrient release. The contents of ammonium and nitrate, urease, invertase, and nitrate reductase in rhizosphere soil of rice under LN treatment were determined. The results showed that the activities of urease, invertase, and nitrate reductase in rhizosphere soil of RC were significantly higher than the WT (Table 5). The content of ammonium in rhizosphere soil of RC was significantly lower than that of WT and the difference was not significant

### 2.5. The Influx and Efflux of Ca^2+^, NO_3_^−^, and NH_4_^+^ in WT and RC

**Figure 7:**
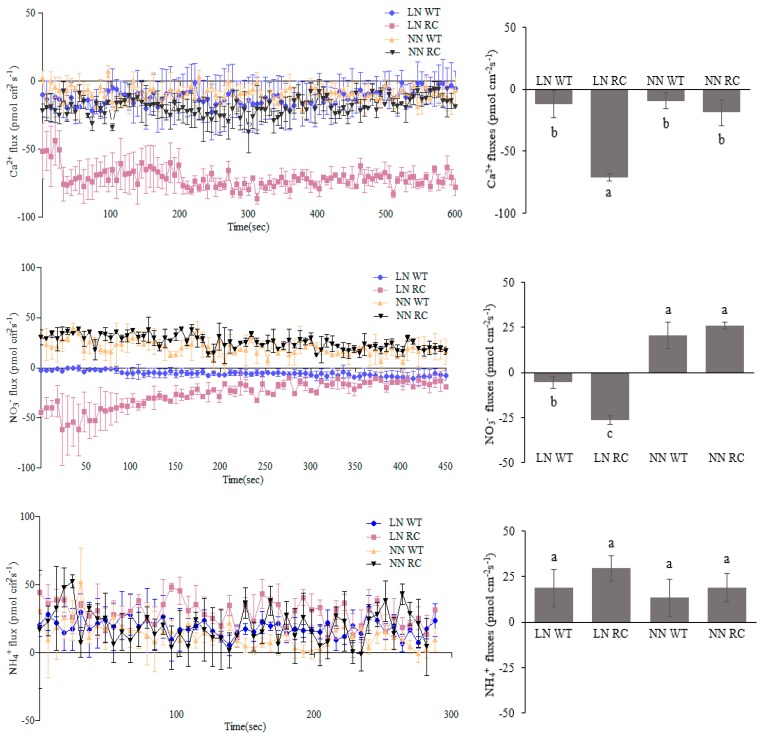
The Ca^2+^,NO_3_^−^, and NH_4_^+^ fluxes of rice root under different N treatments. Left panel represents the flux rate in real time (vertical bars shows the standard erors). Right panel graph represents the total (average) influx and efflux rate. Different small letters beyond the column mean significant difference (*p* < 0.05) calculated by LSD’s test.

Calcium ion uptake in roots of RC increased significantly under LN condition, but there was no significant difference under NN condition (Figure 7). The NO_3_^−^ uptake in roots of RC increased under LN condition, while the roots of both RC and WT showed efflux under NN condition, but there was no significant difference between them. NH_4_^+^ showed efflux, and there was no significant difference between RC and WT. The results showed that the enhanced expression of *CIPK2* in roots promoted the uptake of NO_3_^−^ by roots under LN conditions, which was consistent with the enhanced nitrification in the rhizosphere of RC.

## 3. Discussion

N is an essential factor related to crop yield and quality. For attaining high crop productivity, excessive amounts of N fertilizers are applied in rice production, resulting in low utilization rate. Hence, improving rice NUE is of immense significance for achieving high yielding and environmentally friendly rice production. In this study, we developed a transgenic rice line with an overexpressing *OsCIPK2* gene. The transgenic line performed better than WT under limited N supply. Further, we analyzed rice physiological, microbial, and soil physicochemical properties to confirm comparative NUE between WT and RC. Until now, *OsCIPK2* gene has not been evaluated for its potential at low N levels in rice. Similarly, microbial communities remained unexplored related to the *OsCIPK2* overexpressing rice line. We evaluated the physiochemical aspects in three life stages of significance to the rice life cycle [30].

NUE is a complex biological process including N assimilation, transport, reuse, and many other physiological processes which can be affected by the interaction between genes and environment. [31]. This could be the reason for non-significant difference in grain and dry matter efficiency, as well as nitrogen harvest index in RC and WT under LN. N uptake by rice coupled with more nutrients transported from root, leaf sheath, and other parts to grains could be the reason for the significant increase in rice yield. It has been observed that N-use efficient cultivars produce higher plant biomass as compared to the inefficient NUE cultivars [32]. Researchers found that overexpression of *OsNRT1.1A* could increase rice yield, especially under low nitrogen conditions. It could increase rice yield and NUE by 60%, and shorten the rice growth period under high nitrogen conditions [15]. This indicated that the uptake and utilization of N in rice is regulated by multiple genes.

The activities of N metabolism-related enzymes (nitrate reductase and glutamine synthase) in roots and leaves of transgenic rice increased significantly under the condition of LN (Figure 5). This indicates possibility of better abiotic stress resistance, as glutamine serves as a signaling molecule to *CIPK* gene family for activating response against abiotic stress [33,34]. Besides this, the nutritional metabolism of RC was more enhanced than WT under LN, which was positively supported by increased SPAD value and photosynthetic rate. There was no significant difference between RC rice and WT rice under NN conditions in many analyzed parameters. This could be due to the surplus N causing the reduction to the NUE; excess amount of NUE could cause lodging leading to less yield [35]. Our support for this view comes from previous studies indicating genetic variability as a significant factor in regulating NUE [36]. Wang et al. [37] also showed that *CIPK8* and *CIPK23* in *Arabidopsis thaliana* were induced by NO_3_^−^ regulated nitrate transporter activity, which is in line with our study. Hence, it is inferred that *CIPK2* have involvement in signaling and regulation of N uptake related genes in rice, but how *CIPK2* regulates downstream genes in rice needs further exploration.

Another important aspect related to a plant is its microbial community, which is also known as the second genome of the plant [38,39]. Rhizosphere microorganisms are closely related to N uptake, growth, and development of rice [40,41]. Microbial community structure during the heading stage is closely associated with the rice yield [42]. We studied changes of bacterial community in rhizosphere (R), rhizoplane (RP), and endosphere (ES) of RC and WT at the heading stage. The analysis of the bacterial community showed that the diversity of the bacterial community decreased successively from R > RP > ES in LN and NN treatment. This points out that rice roots were selective to colonization of rhizosphere microorganisms [43].

The abundance of phyla showed variation under the different N levels. The main phyla in the roots were Proteobacteria and Cyanobacteria. The bacteria belonging to these phyla are specifically involved in the carbon and N cycles [43]. The bacterial communities in R and RP of RC consisted of Proteobacteria and Acidobacteria. Just opposite to the WT, Acidobacteria abundance enhanced in R and RP of RC. Acidobacteria population usually is ubiquitous in soil and many members take part in the assimilatory N metabolism, nitrification, and N fixation [44]. We found decreased Acidobacteria abundance and Gemmatimonadetes abundance decreased from the R to ES, which is also supported by the previous research, suggesting that plants have the potential to enrich or deplete certain bacteria by different mechanisms [45]. We found similar results that the soil of a non-fertilized paddy field had high N fixation activity, which could promote rice growth, and diversified the N-fixing microorganisms significantly as compared to fertilized soil [46]. Microbial diversity in a plant’s rhizosphere is known to play various beneficial roles by degrading different organic substances and uplifting plant health, by playing antagonistically against harmful microbial communities [22]. According to our bacterial functional analysis there was a higher abundance of bacteria that play a role in the health of plants and soil in RC under LN. All together, these microbial features have involvement in enhanced root activity and root development for better resistance to stresses [43].

The qPCR was used to determine significant changes in N cycle-related microorganisms under LN. The gene *nifH* is most commonly used to determine the performance of N fixing bacteria [47]. Nitrification, a conversion of NH_4_^+^ or NH_3_ to NO_3_^−^ by the oxidation of NO_3_^−^, is vital for global N cycling. We found that copies of *nifH* and *amoA* genes, involved in nitrification, increased significantly in rhizosphere soil of RC under LN. This shows that the nitrification and N fixation in the rhizosphere of RC in LN were significantly enhanced. The gene *amo* for ammonia monooxygenase, found in AOA and AOB, is the rate-determining step during the microbial nitrification process [48]. Our results further confirmed the increase of AOB and AOA in the rhizosphere of RC, indicative of enhancement of N transformation and related N cycle under LN.

Assessment of soil enzymes can be used to investigate ongoing biochemical activities for estimating soil strength [49]. In our study nitrate reductase (NR), urease, and invertase activities were higher than WT under LN. NR can play a crucial role in generating nitric oxide (NO), which could play a role in improving N uptake by regulating the initiation of lateral roots [50]. Moreover, the dependency of the root to utilize NO_3_^−^ efficiently is related to complex genetic mechanisms linked to morphological and physiological features of plants [51,52,53]. The soil urease is regarded pivotal for its role in hydrolysis of urea to NH_4_^+^, a vital source of N for plant [54]. All these factors play a major role in the N availability to RC under LN. Under NN conditions, soil N supply was sufficient, the abundance of nitrogen cycle-related bacteria was relatively low, and there was little difference between the rhizosphere bacterial communities of RC and WT.

Because NO_3_^−^ was absorbed by the roots immediately after the formation in the rhizosphere, the amount of NO_3_^−^ detected in the rhizosphere was low, and the change in NH_4_^+^ content might be related to the competitive absorption of NH_4_^+^ by rice roots. Non-invasive micro-test technique (NMT) analysis depicted that the absorption of Ca^2+^ and NO_3_^−^ ions increased in RC under LN condition, which further indicated that the increase of Fe(II) oxidizing bacteria coupled with NO_3_^−^ reduction in roots of RC was related to the increase of NO_3_^−^ absorption, and that the reduction of NO_3_^−^ by bacteria further promoted the assimilation of NO_3_^−^ in rice. CDPK and CIPK are both protein kinases which require Ca^2+^ as a prerequisite to activate and start the process of NO_3_^−^ sensing [55,56]. In agricultural soil, plants usually have a major source of N in the form NO_3_^−^. As a signaling compound, NO_3_^−^ can regulate various genes related to plants [57]. We demonstrated that the root length, root surface area, and total root area of RC were significantly increased. Moreover, root activity was also significantly increased leading to increased root nutrient uptake surface area, which ultimately promoted root uptake of N and other nutrients, thus could be the reason for the better shoot growth. As a signal substance, NO_3_^−^ could regulate plant root growth. Kronzucker and colleagues confirmed the incremented absorption of NH_4_^+^ ions in rice due to presence of NO_3_^−^ [58].

## 4. Methods

### 4.1. Plant Material and Transgenic Line Generation

We took rice line Kitaake (*Oryza sativa* L. ssp. *Japonica*) as wild-type and transgenic CIPK2 line (counterpart of wild type) in which the *CIPK2* gene (LOC4344287) was transformed from *Arabidopsis thaliana*, overexpressing the *CIPK2* (NP_196324.1). The line was generated the same as previously by our group [36]. Briefly the p3301 vector was used under the control of the cauliflower mosaic virus promoter (CaMV), used for the purpose of cloning the full length open reading frame (ORF) of *CIPK2* (GenBank accession no. LOC4344287). CaMV 35S was equipped with the FLAG Octapeptide tag. Immature rice embryos from mature seeds were transformed via agrobacterium by following the protocol from previous research [59]. Hygromycin-resistant transformed cells obtained from these tissues were selected for the regeneration of the transgenic plant, which were further verified by FLAG-tag assay and qPCR.

### 4.2. Verification of CIPK2 Gene Overexpression in Rice Roots Thorough qPCR and Western Blot

#### 4.2.1. qPCR

Total RNA was extracted from roots and leaves of RC and WT by using EASYspin Plus Complex Plant RNA extraction kit (Aidlab, Beijing, China) and extracted RNA was used as template. RNA concentration was determined by Infinite M200 (TECAN), and RNA samples were diluted according to RNA concentration before reverse transcription. We synthesized cDNA by using TIANscript RT Kit (Tiangen, Beijing, China) following the manual’s instruction. Quantitative fluorescence polymerase chain reaction (qPCR) using Bestar SybrGreen qPCR Mastermix (DBI Bioscience, Shanghai, China) kit was conducted, using synthesized cDNA as template. We designed the qPCR primers with Primer Premier 5.0 by finding the sequence of *OsCIPK2* and *β-actin* (internal control) genes from the NCBI database. Primers were synthesized by Shanghai Shengsheng Industrial and Biological Co., Ltd. For primers sequence and PCR conditions see Table S1.

#### 4.2.2. Western Blot

RC and WT rice roots and leaves were used for this process. The extracted protein was detected by SDS-PAGE electrophoresis, and the protein concentration was determined by Braford method [60]. The concentration of each sample was diluted to 5–10 mg/mL. According to the FLAG-tag of CIPK2 protein in rice roots, the expression of CIPK2 fusion protein was verified by Western blot using FLAG-tag antibody. The samples were taken for SDS-PAGE at 30 mg. FLAG-tag Polyclonal Antibody (Proteintech Group, Rosemont, IL, USA) was used as the first antibody and Goat Anti-Rabbit IgG (H + L) (Vazyme biotech, Nanjing, China) was used as the second antibody to carry out Western blot following the published paper [36].

### 4.3. Rice Bucket Planting with Different N Treatments

The bucket experiment was carried out in a greenhouse at the experimental base of College of Crop Sciences, Fujian Agricultural and Forestry University, Fujian, Fuzhou, China (26°09’N, 119°23’E) from April to August 2018, with mean temperature in the range of 15 to 34 °C. Plastic buckets were used for cultivation. The bucket height was 0.3 m, and the upper and bottom diameters were 0.3 and 0.23 m, respectively. The soil was taken from the experimental base of College of Crop Sciences, Fujian Agricultural and Forestry University. Sterilized rice seeds were taken and soaked in distilled water, for about one day (24 h) at 25 °C, and later put at 37 °C for 48 h in incubator. Germinated seeds of almost similar sizes were sown to attain the uniform seedling size. The obtained seedlings were transplanted in plastic buckets containing 12 kg soil. The soil texture was sandy loam with total nitrogen content of 1.03 g/kg, available phosphorus 10.2 mg/kg, and the available potassium 71.45 mg/kg. Whereas, the organic matter was 25.81 g/kg. On April 15, 4 plants were planted per bucket. The recommended dose level of N in Fuzhou is 225 kg ha^−1^ control (NN) and LN was without any N fertilizer. Phosphorus was applied as the base fertilizer and potassium as the top dressing, at the rate of 112.5 kg ha^−1^ (P_2_O_5_), and 180 kg ha^−1^ (K_2_O) converted to the amounts per barrel, respectively.

#### 4.3.1. Determination of Rice Plant Nitrogen and Nitrogen Utilization Ratio

One representative rice plant/bucket was taken one day before harvesting, with 3 replications. After washing, the roots, stems, leaves, and panicle were separated. The plants were initially heated at 105 °C for 30 min to deactivate enzymes instantly and then oven dried at 80 °C to attain constant weight. After weighing, each part was ground, 0.5 g of which was digested with concentrated H_2_SO_4_-H_2_O_2_. The total N content of each part was determined by SMARTCHEM200 (Rome, Italy), and the nitrogen content of the plant was calculated. NUE was calculated according to the formulas in Table S3 [7].

#### 4.3.2. Physiological Parameters

After transplanting, the number of tillers of all similarly grown rice plants was recorded after every 10 days until the number of tillers no longer increased, and the average number of tillers was calculated. At tillering, heading, and maturity stages, 6 plants from 6 buckets with similar growth were selected for each treatment. To measure the chlorophyll content the readings of upper, middle, and lower segments of flag leaves were measured by SPAD (soil–plant analyses development) 502 chlorophyll meter at 10:00–11:00 a.m. on sunny days, and the average values were recorded following the method by Xiong et al. [61]. The net photosynthetic rate of the flag leaf was measured by LI-6400 portable photosynthetic instrument (LI-COR, Lincoln, NE, USA). Three representative plants were selected at tillering stage, heading stage, and maturity stage. Roots, stems, leaves, and ears were separated and dried at 105 °C for 30 min and then temperature was set at 80 °C until constant weight was obtained. After cooling, it was weighed and the root:shoot ratio was calculated in total after 3 replications. Root activity was determined by TTC (triphenyl tetrazolium chloride) method [62]. Three representative plants were sampled at tillering, heading, and maturity stages. After carefully digging out roots, the roots were washed with running water. The roots were cut off with scissors, and the root activity of fresh roots was determined immediately. After carefully digging out the whole root system, the surface attachments were washed with running water. After cutting all the roots, digitized scanning was carried out with scanner (EPSON, Suwa, Japan). Then the length of adventitious roots, the length of subdivided roots, the length of thick branched roots, the total root surface area, and the total root body were analyzed by root morphology analysis software WinRHIZO 2013 [63]. The panicle formation of all plants with similar growth potential was recorded at maturity stage. The average effective panicle number was calculated. Five representative rice plants were selected for each treatment per bucket. The number of grains per panicle and the number of real grains were determined, and the seed setting rate, 1000 grain weight, and yield were calculated.

#### 4.3.3. Determination of Rice-Related Enzymes in the Rice

The activities of glutamine synthase (GS) and nitrate reductase (NR) in rice leaves and roots were measured at tillering, heading, and maturing stages, respectively. GS and NR were determined by using the protocol mentioned in previous research [64].

### 4.4. Diversity of Bacterial Communities in Rhizosphere, Rhizoplane, and Endosphere of Rice

#### 4.4.1. Sample Collection

The method of collecting microbial samples in rice rhizosphere soil, rhizoplane, and endosphere refers to [43]. Briefly, at heading stage, one rice plant was sampled from each of the 3 repetitions. Excess soil was removed by shaking vigorously while leaving about 1 mm clinging to the root. The roots was put into a sterile tissue culture bottle containing 50 mL phosphate buffer (PBS), stirred with sterile tweezers to wash down the rhizosphere soil. The root system was taken out and part of the buffer was centrifuged in a centrifugal tube at 4 °C for 30 s. The supernatant was removed and the soil was precipitated as a rhizosphere sample. The extracted roots were placed in a new centrifugal tube with 30 mL PBS buffer. The centrifugal tube was placed in an ultrasonic oscillator for 30 s (working frequency 40 KHz, ultrasonic power 200 W, Shumei Kunshan). The roots were taken out and put into the new PBS buffer. The centrifugal tube was centrifuged at 4 °C for 30 s at 10,000 G. The supernatant was removed and the remaining precipitated portion was kept as a rhizoplane sample. The same roots were further washed down by ultrasound twice to ensure that the microorganisms on the root surface are washed away. The roots were preserved at –80 °C until further processing. DNA was extracted from all samples on the day of sampling.

#### 4.4.2. Microbial DNA Extraction

DNA of different rhizo-compartments (rhizosphere soil, rhizoplane, and endosphere) was extracted through soil genomic DNA extraction kit BioFast Soil Genomic DNA Extraction Kit according to the manufacturer’s instructions. Then all the DNA samples were subjected to gel electrophoresis and further purification, using Universal DNA Purification Kits according to the manufacturer’s instructions (Tiangen Biotech Co., Ltd., Beijing, China). DNA was quantified by using Nanodrop (Thermo Fisher Scientific, Waltham, MA, USA) before being stored at −20 °C for further molecular analysis.

#### 4.4.3. Bacterium 16S rRNA Gene V3–V4 Region Amplification, Quantification, and Sequencing

The concentration of DNA was detected by spectrophotometer Nanodrop 2000 C Spectrophotometer (Thermo Scientific, Waltham, MS, USA). A total of 30 ng of sample was taken for PCR amplification. 16S rRNA gene V3–V4 region was amplified with barcode primers 338F (ACTCCTACGGAGGCAGCAG) and 806R (GGACTACHVGGTWTCTAAT). TransGen Fast pfu DNA polymerase was used in the PCR reaction (Transgen Biotech, Beijing, China). All polymerase chain reaction (PCR) reactions were carried out using PhusionR High-Fidelity PCR Master Mix (New England Biolabs, Ipswich, MS, USA. ). Using 3 replicates of each sample, the PCR products of the same sample were mixed with 2% agarose gel electrophoresis, and the PCR products were recovered by using AxyPrep DNA Gel Recovery Kit (AXYGEN, Union City, CA, USA), Tris-HCl eluted, and 2% agarose electrophoresis. The PCR products were quantified by QuantiFluor ST Blue Fluorescence Quantitative System (Promega, Madison, WI, USA) and then mixed in proportion according to the requirements of each sample. The library was constructed by using TruSeq DNA PCR-Free Sample Preparation Kit (Ilumina, San Diego, CA, USA) and then sequenced by Illumina Miseq (Ilumina, San Diego, CA, USA).

#### 4.4.4. Statistical and Sequencing Data Analysis

After removing barcode and primer sequence, raw_tags of original tags data was subjected to splicing by FLASH (Johns Hopkins University School of Medicine, Baltimore, MD, USA). After further removing chimeras and short sequences, raw_tags can get clean_tags of high quality sequence. OTU clustering [65] (excluding single sequence) was carried out by using usearch V8.1.1861 according to 97% similarity sequence, and representative sequences of OTUs were selected. Rarefaction curve analysis was performed using 97% similarity OTU [65], and the sequences was randomly sampled to construct a rarefaction curve with the number of sequences drawn and the number of OTUs they can represent. With the help of rarefaction curve, the sequencing depth of the sample was obtained (Figure S8). RDP Classifier algorithm and Silva database [66] (Release 119) were used to compare and analyze representative sequences of OTUs, and species information of OTUs community was annotated at various levels (phylum, class, order, family, genus, species). The OTUs abundance in each sample was homogenized, and then alpha diversity and beta diversity were analyzed. The observed_species, Chao1, shannon, good’scoverage, and PD_whole_tree indices were calculated by using Qiime software (Version 1.8, Caporaso labs, Flagstaff, AZ, USA). The distribution curves were drawn by and the differences among groups of alpha diversity indices were analyzed by using R (Version 2.15.3, Foundation for Statistical Computing, Vienna, Austria). The function of microorganisms was predicted by FAPROTAX database and python 2.7 (Python Software Foundation, Delaware, USA). The statistical analysis and drawing of PCA were carried out by the R language program R (Version 2.15.3, Foundation for Statistical Computing, Vienna, Austria).

### 4.5. Quantitative Analysis of N Cycle Related Genes

Rhizosphere nitrogen cycle related genes of *nifH, narG, nirK*, *nirS, nosZ, amoA* (AOA), and *amoA* (AOB) were analyzed using Bestar SybrGreen qPCR Mastermix (DBI Bioscience, Shanghai, China). The reaction was amplified by Bio-Rad CFX Connect Real-time PCR (Bio-Rad, Hercules, CA, USA). The plasmids containing the target gene were diluted to 1, 0.5, 0.1, 0.05, 0.01, 0.005, 0.001, 0.0005 ng/ L, and the sample DNA was diluted to 20 ng/µL. The 15 µL reaction system consists of 7.5 µL 2 × qPCR master mix, 0.6 µL upstream and downstream primers, 1 µL template DNA, and 5.3 µL sterile water. Primer sequences and amplification procedures are as follows: *nosZ*, *nirS*, *narG,* and *nirK* genes were amplified by touchdown PCR, and then pre-denatured for six cycles. The annealing temperature was reduced by 1 °C per cycle. The melting curve was set with reference to the Bio-Rad CFX Connect Real-Time PCR. The standard curve was established according to the results of different concentrations of plasmid PCR, and the number of gene copies in each sample was calculated. Conditions and primer sequences are mentioned in Table S4. The number of plasmid DNA copies was calculated according to the following formula: Plasmid DNA copy number (µL^−1^) = 6.02 × 10^23^ is × plasmid DNA concentration (ng/L)/plasmid DNA molecule amount × 10^9^.

### 4.6. Soil Physicochemical Properties and Enzyme Assays

The available and total amounts of main soil nutrients, such as nitrogen, phosphorus, and potassium, were measured using the methods described by [66,67]. Soil urease activity was determined by incubating 5 g soil with 30 mL of extracting solution at 37 °C for 24 h. The formation of ammonium was measured spectrophotometrically at 578 nm [68]. Soil invertase activity was determined by incubating 5 g soil with 15 mL of 8% sucrose solution at 37 °C for 24 h. The suspension reacted with 3,5-Dinitrosalicylic acid and absorbance was measured at 508 nm [68].

### 4.7. Determination of Ion Flux Rate, Sample Preparation, and Determination Method

Non-invasive micro-measurement system (NMT100-SIM-YG; Younger, Falmouth, MS, USA) was used to measure ion flow velocity in rice roots. Non-invasive micro-measurement technology used specific ion/molecule selective microelectrodes and computer control. Under the condition of no contact with the measured materials, real-time information of ion/molecule flow direction and velocity in and out of the measured materials was obtained. Two nitrogen concentrations were set, the normal nitrogen concentration was 1.44 mM NH_4_NO_3_, and the low nitrogen concentration was 0.24 mMNH_4_NO_3_. The flux of Ca^2+^, NO_3_^−^, NH_4_^+^ in rice root tips were measured by non-invasive micrometer system (NMT) when the seedlings grew to the three-leaves stage [69]. The corresponding electrodes were purchased from Xuyue Technology Co., Ltd (Beijing, China).

Before determination, the prepared electrodes were calibrated with corresponding standard solution (Ca^2+^: CaCl_2_ 0.05, 0.5 mmol/L; NO_3_^−^: KNO_3_ 0.05, 0.5 mmol/L; NH_4_^+^: NH_4_Cl 0.05, 0.5 mmol/L). The prepared rice root system was washed clean with distilled water and put into the corresponding test solution to equilibrate for 30 min (Ca^2+^: 0.1 mmol/L KCl, 0.1 mmol/L CaCl_2_, 0.1 mmol/L MgCl_2_, 0.5 mol/L NaCl, 0.3 mmol/L MES, 0.2 mmol/L Na_2_SO_4_, pH 6.0; NO_3_^−^: 0.1 mmol/L NH_4_NO_3_, 0.1 mmol/L NH_4_NO_3_, 0.1 mmol/L KCl, 0.1 mmol/L KCl, 0.1 mmol/L KCl_2_, 0.3 mmol/CaCl_2_, 0.3 mmol/L MgCl_2_, 0.3 mmol/NH_4_^+^: 0.1 mmol/L NH_4_NO_3_, 0.1 mmol/L CaCl_2_, 0.3 mmol/L MES, pH 6.0), In order to reduce the influence of exoplasmic ion release from roots on the determination results. The balanced apex was immersed in a culture dish filled with fresh test solution, fixed with small stones, and then the ionic changes of the apex were measured with the corresponding ion-selective microelectrodes. The tip of the electrode was adjusted to be as close as possible to the root surface, and the starting point was the reciprocating measurement. The distance of each movement of the electrode was 30 μm and the frequency of movement was 6 s. The voltage difference between the two points was measured. Each sample was continuously measured for about 10 min and repeated three times.

#### Data processing

The original data was converted by Agflux software (Xuyue Technology Co., Ltd., Beijing, China) and the final ion flux rate was obtained. The data was analyzed and mapped by Excel 2016 (Microsoft Corporation, Redmond, WA, USA) and SPSS 13.0 (IBM Corporation, Armonk, NY, USA).

## 5. Conclusions

Nitrogen is one of the most important factors regarding crop production. To achieve high yield, farmers use high doses of N fertilizer, which causes environmental pollution and less profit. To alleviate this situation, innovative yet efficient strategies are needed now more than ever. The development of NUE crops are needed to sustain higher yields on low N consumption, which can be beneficial for both the environment and farmers. In this study we used isogenic lines of Kitaake (WT) and its overexpressing *CIPK2* gene line (RC). We provided evidence about NUE capability of RC. The *OsCIPK2* exhibited significantly better physiological parameters, such as photosynthesis, chlorophyll content, N utilization, yield components, etc. Moreover, enzymatic and qPCR data for N cycle comparatively indicates positive involvement of *CIPK2*, linked to enhanced N cycling. Rhizo-compartmental high-throughput microbial community analysis also revealed microbial diversity in the RC under LN condition, which is usually associated with the healthy growth of plants. Collectively, our data conclude that *OsCIPK2* is a potential candidate for further studies regarding the molecular mechanisms of the involvement of *CIPK2* in the better NUE of rice, and the link of *CIPK2* in the recruitment of beneficial microbial communities by rice.

## Figures and Tables

**Table 1:** Yield and yield components of rice under different nitrogen treatments in pots.

Treatment	Genotype	Valid Panicles (pot^−1^)	Spikelets Per Panicle	Seed Setting Rate (%)	1000 Grain Weight (g)	Grain Yield (g/pot^−1^)
LN	WT	10.68c	37b	97.01a	25a	10.16c
	RC	14.67b	43.25a	82.08c	25.28a	13.75b
NN	WT	44a	38.95a	87.5b	24.72a	37.61a
	RC	44.67a	38.46a	89.82b	24.63a	37.43a

LN—no nitrogen, NN—normal nitrogen, WT—wild type, and RC—transgenic rice. Different small letters in the same column represents significant difference (*p* < 0.05) calculated by LSD’s test.

**Table 2:** N-use efficiency of rice under different nitrogen treatments in buckets.

Treatment	Genotype	N Absorption (mg/plant)	Dry Matter Production Efficiency (BNUE)	Grain Production Efficiency (GNUE)	N Harvest Index (NHI)	Physiological Efficiency (PE)	Agronomic Utilization Rate (AE)	Absorption Utilization Rate (RE)
LN	WT	42.28c	128.59a	63.14a	0.65a	-	-	-
	RC	58.56b	128.31a	62.01a	0.62b	-	-	-
NN	WT	190.56a	93.65b	50.29b	0.49c	46.26a	13.26a	28.68a
	RC	195.21a	94.02b	50.37b	0.48c	44.42b	11.74b	26.43b

LN—no nitrogen, NN—normal nitrogen, WT—wild type, and RC—transgenic rice. Different small letters in the same column represent significant difference (*p* < 0.05) calculated by LSD’s test.

**Table 3:** Diversity index of bacterial communities in rice rhizo-compartments.

Treatment	Sample	Observed_Species	Chao1	Shannon	PD_Whole_Tree
No nitrogen	WS-L	1833.97b	2258.28a	9.3032ab	151.27b
	RS-L	1906.87a	2327.28a	9.4471a	159.03a
	WP-L	1721.50c	2140.65b	9.0611b	144.92c
	RP-L	1842.43b	2296.41a	9.2915ab	156.69a
	WE-L	672.13d	1085.61c	3.6811c	75.16d
	RE-L	645.20d	1034.97c	3.5526c	74.35d
Normal nitrogen	WS-N	2119.53a	2829.45a	9.47a	182.14a
	RS-N	2168.83a	2894.73a	9.47a	190.34a
	WP-N	2112.13a	2799.20a	9.47a	183.68a
	RP-N	2006.36a	2719.17a	9.24a	178.34a
	WE-N	764.56b	1362.97b	3.56b	91.1b
	RE-N	681.55b	1219.97b	2.94b	85.56b

WS-L, WP-L, WE-L represents wild-type rhizosphere, wild-type rhizoplane, wild-type endosphere under no nitrogen, respectively. RS-L, RP-L, RE-L represents transgenic line rhizosphere, transgenic line rhizoplane, transgenic line endosphere under no nitrogen. WS-N, WP-N, WE-N represents wild-type rhizosphere, wild-type rhizoplane, wild-type endosphere under normal nitrogen, respectively. RS-N, RP-N, RE-N represents transgenic line rhizosphere, transgenic line rhizoplane, transgenic line endosphere under normal nitrogen, respectively. Different small letters in the same column represent significant difference level (*p* < 0.05,) determined by LSD’s test.

**Table 4:** Copy numbers of N cycle related genes in rice rhizosphere soil under no nitrogen treatment in buckets.

Gene	*nifH*	*amoA* (AOA)	*amoA* (AOB)	*narG*	*nirK*	*nosZ*	*nirS*
WT	7.29 × 10^12^b	4.99 × 10^7^b	4.20 × 10^6^b	1.69 × 10^6^a	7.59 ×1 0^4^a	1.77 × 10^6^a	9.01 × 10^7^b
RC	8.54 × 10^12^a	5.60 × 10^7^a	4.75 × 10^6^a	1.83 × 10^6^a	3.50 × 10^4^b	2.83 × 10^6^a	12.84 × 10^7^a

Different letters show significant differences (*p* < 0.05) determined by LSD’s test.

**Table 5:** N-related physicochemical properties of rice rhizosphere soil under no nitrogen treatment (LN).

Genotype	NH_4_^+^-N (mg·kg^−1^)	NO_3_^−^-N (mg·kg^−1^)	Urease (mg·g^−1^·24h^−1^)	Invertase (mg·g^−1^·24h^−1^)	Nitrate Reductase (μg NO_2_^−^·g^−1^·24h^−1^)
WT	27.52 ± 0.46a	2.14 ± 0.60a	0.118 ± 0.003b	2.40 ± 0.083b	2.52 ± 0.63b
RC	24.85 ± 1.10b	2.35 ± 0.49a	0.138 ± 0.004a	2.61 ± 0.062a	5.53 ± 0.82a

Different letters in columns show significant differences (*p* < 0.05) determined by LSD’s test. Data are means ± standard errors.

## Supplementary Materials

Supplementary materials can be found at https://www.mdpi.com/1422-0067/20/15/3636/s1. Figure S1. (a) Tillering dynamics of rice under different nitrogen treatments in pots. (b) Effective spike number and ear-bearing tiller percentage of rice under different nitrogen treatments in pots. Figure S2. Rice roots at heading stage under no nitrogen treatment in bucket. Figure S3. Root activity of rice at different growth stages under different nitrogen treatments in bucket. Figure S4. Dry weight and root: shoot ratio of rice at different growth stages. Figure S5. Venn diagram of OTUs in rice rhizo-compartments. Figure S6. The results of principal component analysis of bacterial communities in rhizo-compartments of rice. Figure S7. Heat map of OTUs abundance of different functional bacteria in rhizo-compartments of rice under different nitrogen treatments. Figure S8. Rarefaction curves of rhizosphere, rhizoplane, and endosphere samples of rice. Table S1. Primers and conditions of OsCIPK2 and actin gene. Table S2. Morphological characteristics of roots under different nitrogen treatments in pots. Table S3. Determination of rice plant nitrogen and nitrogen utilization ratio. Table S4. Nitrogen cycle-related gene primer sequences and amplification conditions.
