# Supplementary material for: Exploring the Potential of Overexpressed OsCIPK2 Rice as a Nitrogen Utilization Efficient Crop and Analysis of Its Associated Rhizo-Compartmental Microbial Communities
Source: Int J Mol Sci. 2019 Jul 25;20(15):3636. doi: 10.3390/ijms20153636 (PMC6695771; doi:10.3390/ijms20153636)
Supplement: Supplementary file 1 [file ijms-20-03636-s001.pdf]

International Journal of
Molecular Sciences

MDPI

Supplementary Materials

Exploring the Potential of Overexpressed *OsCIPK2* Rice as a Nitrogen Utilization Efficient Crop and Analysis of Its Associated Rhizo-Compartmental Microbial Communities

Muhammad Umar Khan [1,2,†,*], Penghui Li [2,3,†], Hira Amjad [1,2], Ali Qaiser Khan [4], Yasir Arafat [1,2], Muhammad Waqas [2,3], Zhong Li [1,3], Ali Noman [5], Waqar Islam [6], Linkun Wu [1], Zhixing Zhang [1] and Wenxiong Lin [1,2,3,*]

[1] Fujian Provincial Key Laboratory of Agroecological Processing and Safety Monitoring, College of Life Sciences, Fujian Agriculture and Forestry University, Fuzhou 350002, China
[2] Key Laboratory of Crop Ecology and Molecular Physiology, Fujian Agriculture and Forestry University, Fuzhou 350002, China
[3] Key Laboratory for Genetics, Breeding and Multiple Utilization of Crops, Ministry of Education/College of Crop Sciences, Fujian Agriculture and Forestry University, Fuzhou 350002, China
[4] College of Agriculture, Nanjing Agricultural University, Nanjing 210095, China
[5] Department of Botany, Government College University, Faisalabad 38000, Pakistan
[6] College of Geographical Sciences, Fujian Normal University, Fuzhou 350007, China
* Correspondence: umar.khan018@yahoo.com (M.U.K); lwx@fafu.edu.cn (W.L.); Tel.: +86-591-83737535, (W.L.)
† These authors contributed equally to this work

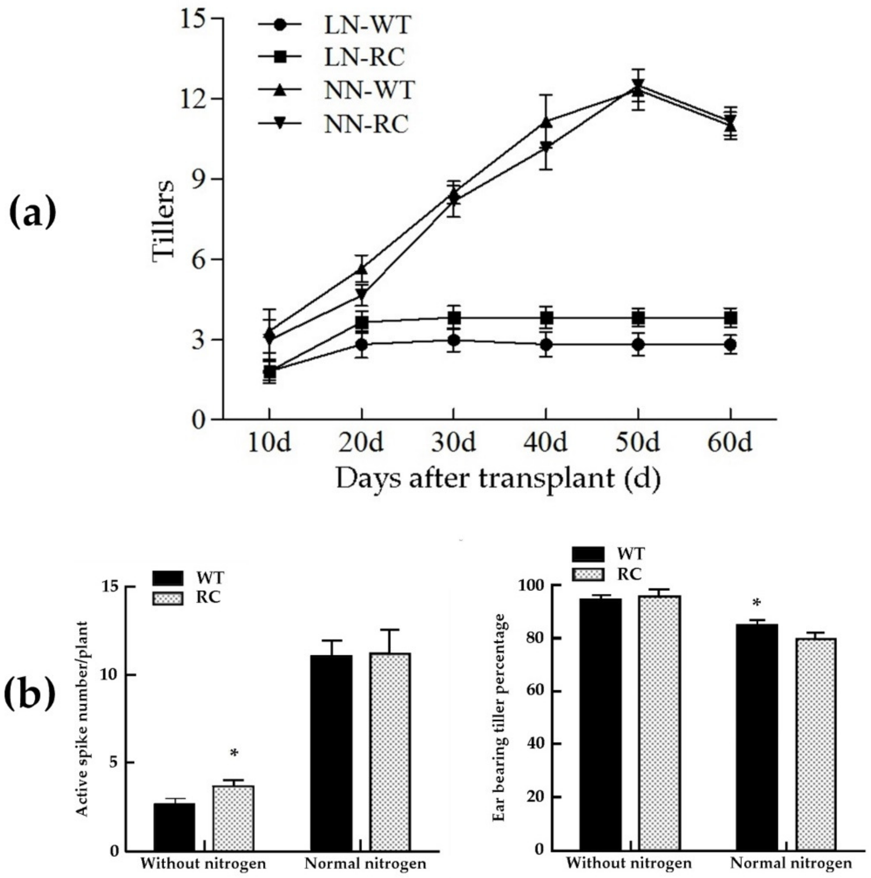

Figure S1. (**a**) Tillering dynamics of rice under different nitrogen treatments in pots. LN –WT: No N Wild type, LN-RC: Zero N Transgenic rice, NN-WT: Normal Nitrogen Wild type and NN-RC: Normal Nitrogen Transgenic rice. (**b**) Effective spike number and ear bearing tiller percentage of rice under different nitrogen treatments in pots. LN: no nitrogen treatment, NN: normal nitrogen treatment. *indicate significant difference at ($p < 0.05$) between different samples in the same treatment calculated by LSD's test.

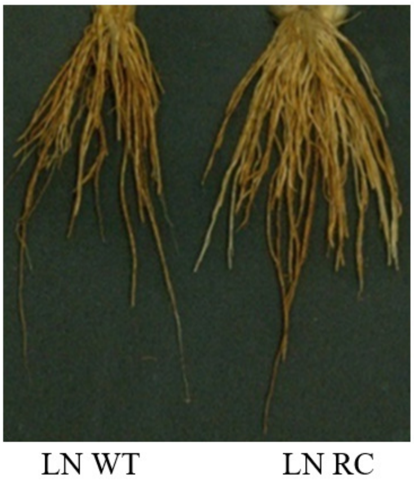

Figure S2. Rice roots at heading stage under no nitrogen treatment in bucket.

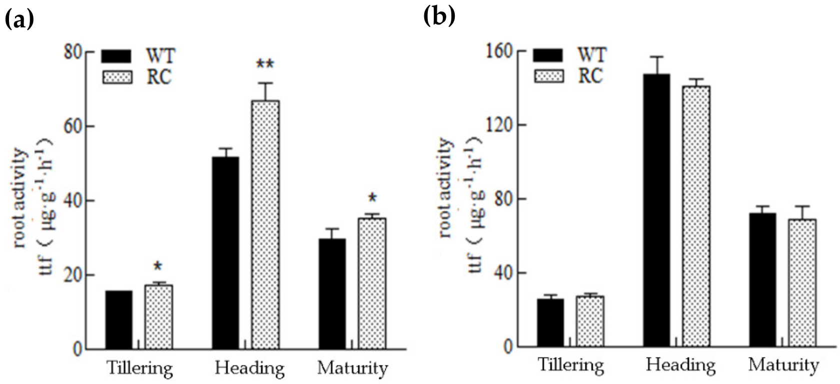

Figure S3. Root activity of rice at different growth stages under different nitrogen treatments in pots **a**: LN, no nitrogen treatment, **b**: NN, normal nitrogen treatment. *, **indicates significant difference ($p < 0.05$) between different samples in the same growth period calculated by LSD's test.

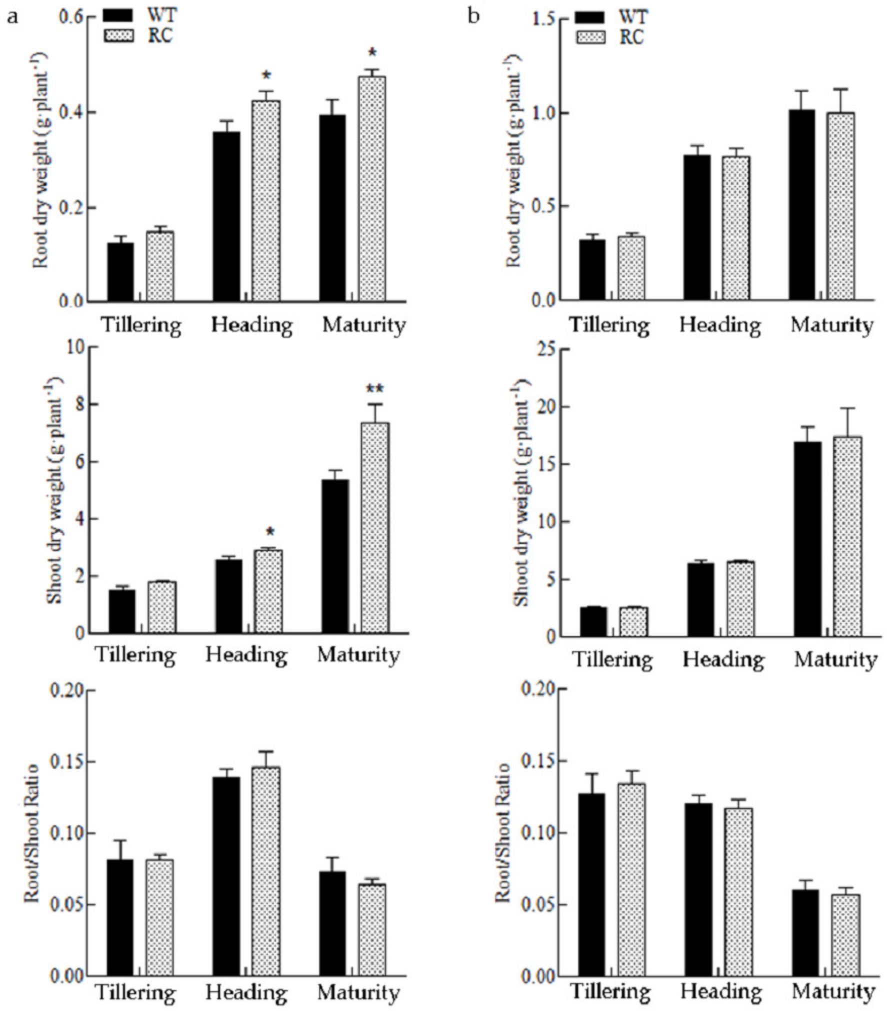

Figure S4. Dry weight and root-shoot ratio of rice at different growth stages. (**a**) no nitrogen treatment, (**b**) normal nitrogen treatment. *, **indicates significant difference ($p < 0.05$) between different samples in the same growth period calculated by LSD's test.

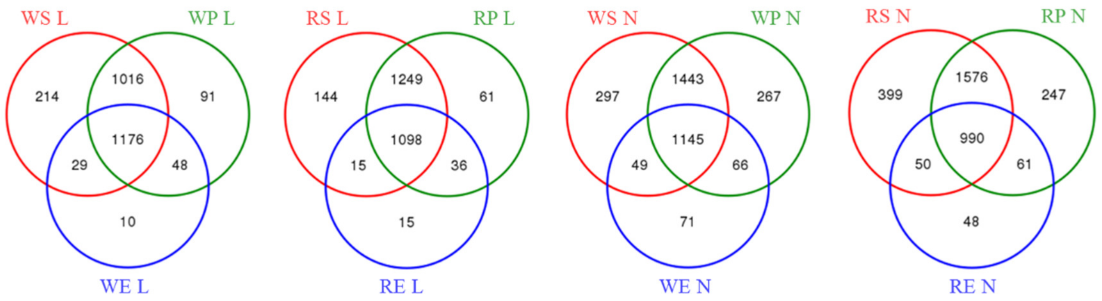

Figure S5. Venn diagram of OTUs in rice rhizo-compartments. WSL, WPL, WE L represents Wild type rhizosphere, wild type rhizoplane, wild endosphere under no nitrogen, respectively. RS L, RP L, RE L represents transgenic line rhizosphere, transgenic line rhizoplane, transgenic line endosphere under no nitrogen. WS N, WP N, WE N represents Wild tye rhizosphere, wild type rhizoplane, wild endosphere under normal nitrogen, respectively. RS N, RP N, RE Nrepresents transgenic line rhizosphere, transgenic line rhizoplane, transgenic line endosphere under normal nitrogen, respectively.

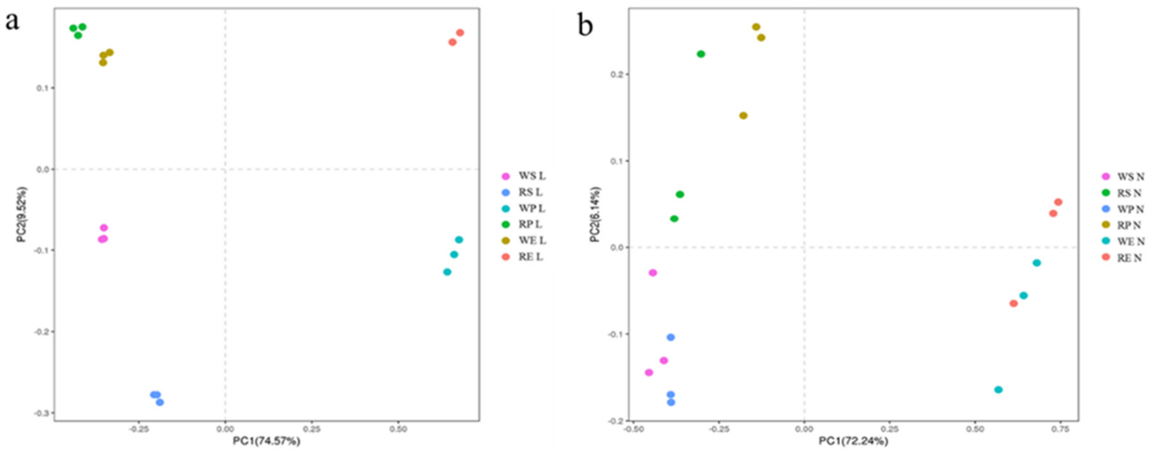

Figure S6. The results of principal component analysis of bacterial communities in rhizo-compartments of rice. WS L, WPL,WE L represents Wild type rhizosphere, wild type rhizoplane, wild endosphere under no nitrogen, respectively. RS L, RP L, RE L represents transgenic line rhizosphere, transgenic line rhizoplane, transgenic line endosphere under no nitrogen. WS N, WP N, WE N represents Wild type rhizosphere, wild type rhizoplane, wild endosphere under normal nitrogen, respectively. RS N, RP N, RE N represents transgenic line rhizosphere, transgenic line rhizoplane, transgenic line endosphere under normal nitrogen, respectively.

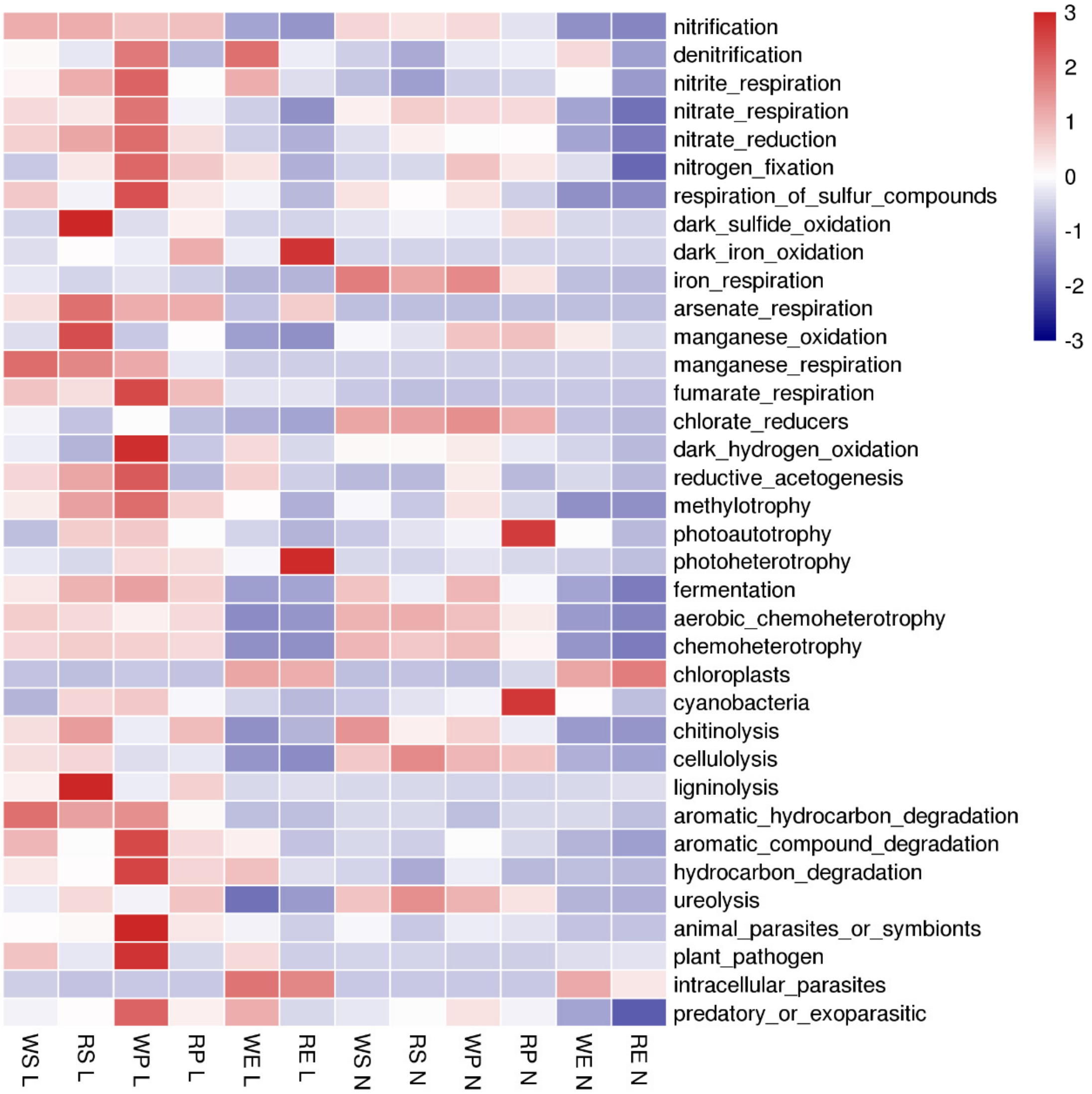

Figure S7. Heat map of OTUs abundance of different functional bacteria in rhizo-compartments of rice under different nitrogen treatments. The heat map shows the Z-score of relative abundance of each functional bacteria, the relative values for bacteria of different function are indicated by color intensity.

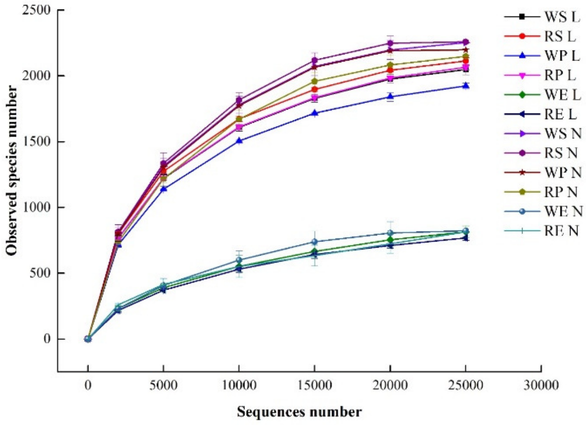

Figure S8. Rarefaction curves of rhizosphere, rhizoplane, endosphere samples of rice. WS, WE, WS respectively represent rhizosphere, rhizoplane, endosphere of WT; RS, RP, RE refers to rhizosphere, rhizoplane, endosphere of root specific expression CIPK2 rice (RC), L represents no nitrogen treatment, N represents normal nitrogen treatment, the same as below.

Table S1. Primers and Conditions *OsCIPK2* and *actin* gene.

**The primer sequence is as follows**	
Actin Forward primer	5'GGAACTGGTATGGTCAAGGC 3'
Actin Reverse primer	3'AGTCTCATGGATAACCGCAG 3'
OsCIPK2 Forward primer	5' GCCCGTAACACCGAGACTT 3'
OsCIPK2 Reverse primer	5' GCCTCACCAACTTCATCACA 3'
**The reaction system is as follows**	
2× qPCR mastermix	7.5μL
Forward primer	0.5μL
Reverse primer	0.5μL
cDNA	0.5μL
Rnasefree water to make up the total system of 15μL	
Actin gene PCR amplification reaction profile: 95°C 5min;	
95°C 15s<br>55°C 20s — 35 cycles<br>72°C 30s	
OsCIPK2 gene PCR amplification reaction profile: 95°C 5min;	
95°C 15s<br>55°C 20s — 35 cycles<br>72°C 30s	

Melting curve analysis refers to the standard set value of Bio-rad CFX Connect (Bio-rad) of PCR instrument. The expression of CIPK2 gene among different rice genotypes was calculated by $2^{-\Delta\Delta ct}$ method.

Table S2. Morphological characteristics of roots under different nitrogen treatments in pots.

Stage	Treatment	Genotype	Adventitious root length(m)	Fine lateral root length(m)	Coarse lateral root length(m)	Total Rhizoplane area(cm^2)	Total root volume (cm^3)
Heading	LN	WT	6.29c	1.08c	0.90c	159.50c	2.627c
		RC	9.05b	2.28b	1.98b	231.18b	2.964b
	NN	WT	17.64a	4.10a	3.12a	452.02a	6.007a
		RC	17.83a	4.19a	3.03a	445.67a	5.750a
Maturity	LN	WT	4.85c	2.04c	0.93b	101.10b	1.012b
		RC	5.91b	2.75b	1.48b	124.58b	1.181b
	NN	WT	14.72a	6.65a	3.37a	356.10a	4.244a
		RC	14.61a	7.18a	3.49a	350.09a	4.068a

Table S3. Rice plant nitrogen and nitrogen utilization ratio.

Biomass nitrogen use efficiency (BNUE) = dry matter accumulation/total nitrogen uptake per plant
Nitrogen grain utilization efficiency (NGUE) = grain yield/total nitrogen uptake per plant
Nitrogen harvest index (NHI) = grain nitrogen accumulation/total plant nitrogen uptake
Nitrogen Physiological Efficiency (PE) = (Grain Yield in Nitrogen-Free Zone) / (Total Nitrogen Uptake in Nitrogen-Free Zone) / (Total Nitrogen Uptake in Nitrogen-Free Zone)
Nitrogen agronomic efficiency (AE) = (grain yield in nitrogen-free area) / nitrogen application rate
Nitrogen recovery efficiency (RE) = (total nitrogen uptake by plants in nitrogen-free zone) / nitrogen application *100

Table S4. Nitrogen cycle related gene primer sequences and amplification conditions.

Gene	Primer Sequences (5′–3′)	conditions
nifH [1]	nifH-F: CCTACGGGAGGCAGCAG<br>nifH-R: ATTACCGCGGCTGCTGGCA	95°C 5min;<br>95°C 45s, 59°C 45s, 72°C 45s; 39 cycles
amoA(AOB) [2]	AOB-F: GGGGTTTCTACTGGTGGT<br>AOB-R: CCCCTCKGSAAAGCCTTCTTC	95°C 15min;<br>94°C 45s, 58°C 45s, 72°C 45s; 39 cycles
amoA(AOA) [3]	AOA-F: ATGGTCTGGCTWAGACG<br>AOA-R: GCCATCCABCKRTANGTCCA	95°C 10min;<br>94°C 45s, 55°C 45s, 72°C 45s; 39 cycles
nosZ [4,5]	noSZ-F: CGCRACGGCAASAAGGTSMS<br>nosZ-R: SGTCAKRTGCAKSGCRTGGCAGAA	95°C 15min;<br>95°C 15s, 65-60°C 30s, 72°C 30s, 80°C 15s, 6 cycles; 95°C 15s, 64°C 30s, 72°C 30s, 80°C 15s; 40 cycles

nirS [5]	nirS-F: AACGYSAAGGARACSGG<br>nirS-F: GASTTCGGRTGSGTCTTSAYGAA	95°C 15min;<br>95°C 15s, 65-60°C 30s, 72°C 30s, 80°C 15s, 6 cycles; 95°C 15s, 63°C 30s, 72°C 30s, 80°C 15s; 40 cycles
narG [6]	narG-F: TAYGTSGGGCAGGARAAACTG<br>narG-R: CGTAGAAGAAGCTGGTGCTGTT	95°C,15min;<br>95°C 15s,65-60°C 30s, 72°C 30s, 80°C 15s, 6 cycles; 95°C 15s, 60°C 30s, 72°C 30s, 80°C 15s; 40 cycles
nirK [7]	nirK-F: ATYGGCGGVCAYGGCGA<br>nirK-R: GCCTCGATCAGRTTRTGGTT	95°C 15min;<br>95°C 15s, 63-58°C 30s, 72°C 30s, 80°C 15s; 6 cycles; 95°C 15s, 60°C 30s, 72°C 30s, 80°C 15s, 40 cycles

1. Van Kessel, C.; Hartley, C., Agricultural management of grain legumes: has it led to an increase in nitrogen fixation? *Field Crops Research* **2000,** 65, (2-3), 165-181.
2. Jensen, E. S.; Hauggaard-Nielsen, H.; Kinane, J.; Andersen, M. K.; Jørnsgaard, B. In *Intercropping–The practical application of diversity, competition, and facilitation in arable and organic cropping systems*, Researching Sustainable Systems 2005. Proceedings of the First Scientific Conference of the International Society of Organic Agricultural Research (ISOFAR). 2005; ISOFAR, Bonn, Germany: 2005; pp 22-25.
3. Zhang, X.-q.; Huang, G.-q.; Bian, X.-m.; Jiang, X.-h.; Zhao, Q., Review of researches on advantages of intercropping. *Asian Agricultural Research* **2012,** 4, (1812-2016-143519), 126.
4. Poggio, S. L., Structure of weed communites occurring in monoculture and intercropping of field pea and barley. *Agriculture Ecosystems & Environment* **2005,** 109, (1), 48-58.
5. Poggio, S. L., Structure of weed communities occurring in monoculture and intercropping of field pea and barley. *Agriculture, ecosystems & environment* **2005,** 109, (1-2), 48-58.
6. Li, L.; Zhang, F.; Li, X.; Christie, P.; Sun, J.; Yang, S.; Tang, C., Interspecific facilitation of nutrient uptake by intercropped maize and faba bean. *Nutrient Cycling in Agroecosystems* **2003,** 65, (1), 61-71.
7. Latif, M.; Mehuys, G.; Mackenzie, A.; Alli, I.; Faris, M., Effects of legumes on soil physical quality in a maize crop. *Plant and Soil* **1992,** 140, (1), 15-23.
